# Development of a robust, field-deployable loop-mediated isothermal amplification (LAMP) assay for specific detection of potato pathogen *Dickeya dianthicola* targeting a unique genomic region

**DOI:** 10.1371/journal.pone.0218868

**Published:** 2019-06-24

**Authors:** Jordie Ocenar, Dario Arizala, Gamze Boluk, Upasana Dhakal, Samudra Gunarathne, Sujan Paudel, Shefali Dobhal, Mohammad Arif

**Affiliations:** 1 Department of Plant and Environmental Protection Sciences, University of Hawaii at Manoa, Honolulu, Hawaii, United States of America; 2 Department of Agriculture, State of Hawaii, Honolulu, Hawaii, United States of America; Oklahoma State University, UNITED STATES

## Abstract

Destructive maceration, a wide host range, and longevity in non-plant substrates has established *Dickeya dianthicola* (blackleg of potato) as a significant threat to potato industries worldwide. To protect these businesses, a specific and sensitive point-of-care *D*. *dianthicola* detection tool is necessary. We have developed a loop-mediated isothermal amplification (LAMP) assay for specific, sensitive, and rapid detection of *D*. *dianthicola*, which can be streamlined for point-of-care use. The developed LAMP assay targets a unique gene, *alcohol dehydrogenase*, of *D*. *dianthicola*. Assay specificity was assessed using strains present in inclusivity (16 *D*. *dianthicola* strains) and exclusivity panels (56 closely related, potato pathogenic, and other bacterial strains). Amplification with strains of inclusivity panel occurred, and cross-reactivity with non-target DNA was not observed. The limit of detection (LOD) was 10 CFU/ml when dilutions were made before isolating the genomic DNA; however, LOD was determined as 1 pg using 10-fold serially diluted *D*. *dianthicola* genomic DNA. Similar LOD of 1 pg was observed when serially diluted target genomic DNA was mixed with host genomic DNA. LOD (1 pg) was also calculated with 10-fold serially diluted synthetic DNA fragments containing primer target sites. Naturally and artificially inoculated plant samples were used for field adaptability tests with the field-deployable Optigene Plant Material Lysis Kit and a heat block (65°C); the results were obtained within 20 minutes. Despite the lack of method precision, no false positives or false negatives were observed. Therefore, with prepared reactions and a steady heat source, this assay can be used for rapid point-of-care detection, which is imperative for quarantine, eradication, disease management, and border protection.

## Introduction

Potato (*Solanum tuberosum*) is the tenth most produced crop in the world [1]. High production may be attributed to its use in multiple industries and ability to grow in diverse climate conditions [2–3]. Under various climates, several bacterial diseases have been reported on potatoes [4–5]. Of the bacterial diseases, soft rot and blackleg of potato caused by *Dickeya dianthicola* is one of the most devastating diseases damaging economies worldwide [6–8].

*D*. *dianthicola* (previously *Erwinia chrysanthemi* pv. *dianthicola; Pectobacterium chrysanthemi* pv. *dianthicola*) is a seed borne phytopathogen [9] and has been detected in the European Union, South America, New Zealand, Western Australia, Japan, and the United States [7, 10, 11]. Throughout these countries, *D*. *dianthicola* has a wide host range [6, 12, 13], but potato is considered the main host [14, 15]. Infected potato plants exhibit wilting, dwarfing, and wet, oozy, black stems [3]. The pathogen is highly virulent and requires less inoculum than *Pectobacterium atrosepticum* and *P*. *carotovorum* to cause severe symptoms in potato [6, 14]. At high temperatures (between 25 and 30°C), *D*. *dianthicola* produces noticeable destruction and decay, but at low temperatures can be visually undetectable. Furthermore, *D*. *dianthicola* can survive in soil and water for several months, increasing the probability of spread and contamination to potential hosts [6, 7, 16]. Consequently, it is important to develop efficient, sensitive, field-ready diagnostic tools for specific detection of *D*. *dianthicola*.

*Dickeya* species can be identified using a series of biochemical tests paired with semi-selective media as well as lateral flow immunoassay [17]; neither method is time efficient or accurate. In contrast, molecular techniques are specific and sensitive for detection and differentiation of *Dickeya* to species [18–21]. Conventional PCR is an important nucleic-acid technique but is not time efficient or field-deployable. The advancements of isothermal methods have provided rapid and sensitive techniques that can be used at point-of-care without the need of special equipment.

Loop-mediated isothermal amplification (LAMP) is a popular isothermal, nucleic acid amplification-based technique used for detection of several plant bacterial pathogens [22–24]. LAMP employs a strand displacing *Bst* polymerase for the million-folds amplification of the target DNA duplex [25]. The reaction uses 4–6 primers to specifically bind to 6–8 specific regions in the target genome [22, 26].

For a LAMP reaction to be highly specific and exclusive, it is imperative to find a unique gene region for primer design [22,23]. The unique regions can be identified by comparative genomic analyses of different strains of the same species and other neighboring species/genera [22, 27–29]. Primer specificity and broad range detection capabilities can be tested against strains of inclusivity and exclusivity panels [30]. If a truly unique region is not used, then the diagnostic tool may produce non-specific results [27, 31]. Overall, determining a sequence unique to a species is the key to developing a successful and robust LAMP diagnostic tool [22, 23].

Numerous chemistries (pyrophosphate turbidity, fluorescence, gel electrophoresis) are available for observing positive LAMP amplification but require special equipment for visualization [32–34]. However, SYBR Green I stain has the advantage of producing a color change that is observable without equipment [22, 23]. When added to a LAMP reaction, results are visible almost immediately, which is useful in time-sensitive situations. Although, a heat block is adequate, several battery-operated portable commercial real-time LAMP instruments, like the Genie II [35] or BioRanger, suitable for field application are available [23, 36].

Currently, LAMP has been developed for the *Dickeya* genus [37]. Nonetheless, a swift, convenient, and reliable diagnostic method is needed for direct identification of the aggressive *D*. *dianthicola*. The purpose of this study was to develop a LAMP reaction for specific, accurate, and rapid detection of *D*. *dianthicola* from infected plant tissues. Potential applications include point-of-care plant disease diagnosis for disease management, field surveys, and biosecurity of agricultural crops.

## Materials and methods

### Ethical statement

No permission was required from government agencies or regulatory bodies to include the infected samples in this study. Endangered or protected species were not collected or used in this study. No samples were collected from endangered or protected field sites.

### Source isolates, plant inoculation and DNA isolation

Sixteen isolates of *D*. *dianthicola* and fifty-six isolates of closely related species and genera were selected for inclusivity and exclusivity panels, respectively (Tables 1 and 2). Strains from worldwide locations and hosts were chosen from the Pacific Bacterial Collection (University of Hawaii at Manoa). Selected bacterial strains were cultured on tetrazolium chloride media (TZC; 5 g peptone, 2.5 g dextrose, 8.5 g agar and 0.5 ml 1% TZC in 500 ml distilled water) at room temperature. Culture plates were incubated at 26 ± 2°C and a single colony of each plate was re-cultured.

**Table 1:** Detailed description of bacterial strains used in the inclusivity panel to validate the developed LAMP assay for specific detection of *Dickeya dianthicola*.

Isolate Code	Original Lab ID	Species Name	Location	Host	LAMP Results	NCBI GenBank Accession Number
A5418	CFBP1200	*Dickeya dianthicola*	UK	*Dianthus caryophyllus*	+	MK208961
A5566	PRI 1363	*D*. *dianthicola*	Netherlands	*Solanum tuberosum*	+	MK208962
A5567	PRI 1370	*D*. *dianthicola*	Netherlands	*S*. *tuberosum*	+	MK208963
A5568	PRI 1372-A	*D*. *dianthicola*	Netherlands	*S*. *tuberosum*	+	MK208964
A5569	PRI 1372-B	*D*. *dianthicola*	Netherlands	*S*. *tuberosum*	+	MK208965
A5570	PRI 1600	*D*. *dianthicola*	Netherlands	*S*. *tuberosum*	+	MK208966
A5572	PRI 1741-B	*D*. *dianthicola*	Netherlands	*S*. *tuberosum*	+	MK208946
A5573	PRI 2114	*D*. *dianthicola*	United Kingdom	*Dianthus caryophyllus*	+	MK208947
A5644	CFBP2015	*D*. *dianthicola*	France	*S*. *tuberosum*	+	MK208951
A5645	CFBP 4155	*D*. *dianthicola*	Netherlands	*Kalanchoe blossfeldiana*	+	MK208952
A6058	CFBP1982	*D*. *dianthicola*	France	*Dahlia* sp.	+	MK208953
A6059	CFBP3706	*D*. *dianthicola*	Switzerland	*Cichorium intybus*	+	MK208955
PL22	GBp1A	*D*. *dianthicola*	Hawaii, USA	*S*. *tuberosum*	+	MK189269
PL23	GBp10B	*D*. *dianthicola*	Hawaii, USA	*S*. *tuberosum*	+	MK189270
PL24	GBp11A	*D*. *dianthicola*	Hawaii, USA	*S*. *tuberosum*	+	MK189271
PL25	GBp21C	*D*. *dianthicola*	Hawaii, USA	*S*. *tuberosum*	+	MK189268

Plus (+) sign indicates positive amplification.

**Table 2:** A detailed description of bacterial strains used in the exclusivity panel to validate the developed LAMP assay for specific detection of *Dickeya dianthicola*.

Isolate Code	Original Lab ID	Species Name	Location	Host	LAMP Results	NCBI GenBank Accession Number
A2961	C58	*Agrobacterium tumefaciens*	New York, USA	*Prunus avium*	–	Not submitted
A6181	CC97	*Bacillus* sp.			–	MK202803
A1838	UC 202.1B	*Candidatus* Pectobacterium maceratum		*Solanum tuberosum*	–	MK189264
A4763	N 7388A	*Clavibacter michiganensis* subsp. *michiganensis*	Morocco	*S*. *lycopersicum*	–	MH560485
A2041	R8	*Clavibacter michiganensis* subsp. *spedonicus*	Denmark	*S*. *tuberosum*	–	MH560493
A5415	CFBP2048	*Dickeya chrysanthemi*	USA	*Chrysanthemum* sp.	–	MH453538
A5641	CFBP 1270	*D*. *chrysanthemi*	Denmark	*Parthenium*	–	MH453539
A6061	CFBP1247	*D*. *dadantii*	USA	*Dieffenbachia picta*	–	MK208957
A5420	CFBP4178	*D*. *dadantii*	Colombia	*Musa paradisiaca*	–	MK208942
A5579	PRI2127	*D*. *dadantii*	Colombia	*M*. *paradisiaca*	–	MK208943
A5643	CFBP 6467	*D*. *dadantii*	Martinique	*Musa* sp.	–	MK208950
A6060	CFBP3698	*D*. *dadantii*	Cuba	*Musa* sp.	–	MK208956
A5416	CFBP1269	*D*. *dadantii*	Comoros	*Perlagonium capitatum*	–	MK208944
A5642	CFBP 3855	*D*. *dadantii*	France	*Saintpaulia*	–	MH453542
A5581	PRI 2187	*D*. *solani*	Israel	*S*. *tuberosum*	–	MH453540
A5582	PRI 2188	*D*. *solani*	Israel	*S*. *tuberosum*	–	MH453541
A5263	1-1A	*D*. *zeae*	Hawaii, USA	*Ananas comosus*	–	MK189272
A5265	1-3A	*D*. *zeae*	Hawaii, USA	*A*. *comosus*	–	MK189273
A5306	3–5	*D*. *zeae*	Hawaii, USA	*A*. *comosus*	–	MK189274
A5423	CFBP6466	*D*. *zeae*	Martinique	*A*. *comosus*	–	MH453536
A6056	3 leaf	*D*. *zeae*	Hawaii, USA	*A*. *comosus*	–	MH453535
A5422	CFBP2052	*D*. *zeae*	USA	*Zea mays*	–	MH453537
A5150		*Enterobacter cloacae*	Hawaii, USA	*Zingiber officinale*	–	MK182852
A5149	B193	*E*. *cloacae*	Hawaii, USA	*Z*. *officinale*	–	MK182850
A1084	QR-6	*Erwinia amylovora*		*Pyrus* sp.	–	MK182851
A5367	4C	*Erwinia* sp.	Hawaii, USA	*Aglaonema* sp.	–	MK243480
A5369	8X	*Erwinia* sp.	Hawaii, USA	*Aglaonema* sp.	–	Not submitted
A3131	ATCC13048	*Klebsiella aerogenes*			–	MK208954
A223	A223-9	*Klebsiella* sp.		*Vanda* sp.	–	MK182842
A5186	ATCC29267	*Pantoea* *cypripedii*	California, USA	*Cypripidium* sp.	–	MK182846
A5513		*P*. *agglomerans*	Hawaii, USA	Ornamental	–	MK182849
A6222	DP138	*P*. *agglomerans*	Wisconsin, USA	*Z*. *mays*	–	MH547382
A1867	F2 c. papaya-purple	*Pantoea* sp.	Hawaii, USA	*Carica papaya*	–	MK182844
A1850	IPM 1260	*Pectobacterium atrosepticum*	Colorado, USA	*S*. *tuberosum*	–	MH453513
A6163	Eca31	*P*. *atrosepticum*	Wisconsin, USA	*S*. *tuberosum*	–	Not submitted
A6167	Ecb6	*P*. *betavasculorum*	California, USA	*Beta vulgaris*	–	MK250994
A3048	E60	*P*. *carotovorum* subsp. *brasiliensis*		*Brassica oleracea*	–	MH453523
A6149	WPP5	*P*. *carotovorum* subsp. *brasiliensis*	Wisconsin, USA	*S*. *tuberosum*	–	MH453522
A4682	9X	*P*. *carotovorum* subsp. *carotovorum*	Hawaii, USA	*Aglaonema* sp.	–	MK208939
A5350	5C	*P*. *carotovorum* subsp. *carotovorum*	Hawaii, USA	*Aglaonema* sp.	–	MK208940
A5352	T-15	*P*. *carotovorum* subsp. *carotovorum*	Hawaii, USA	*Aglaonema* sp.	–	MH453529
A5280	1-#31	*P*. *carotovorum* subsp. *carotovorum*	Hawaii, USA	Irrigation Water	–	MH453512
A6273	BA17	*P*. *carotovorum* subsp. *carotovorum*	Hawaii, USA	*S*. *lycopersicum*	–	MK453527
A2686	E43	*P*. *carotovorum* subsp. *odoriferum*	Hawaii	*B*. *oleracea* var. *capitata*	–	MH453519
A1089	QR-11	*P*. *carotovorum* subsp. *odoriferum*	California, USA	*Capsicum annum*	–	MH453518
A1852	M784	*P*. *parmentieri*	Colorado, USA	*S*. *tuberosum*	–	MH453534
A6159	WPP168	*P*. *parmentieri*	Wisconsin, USA	*S*. *tuberosum*	–	MH453533
PL63	K-G	*P*. *carotovorum* subsp. *brasiliensis*	Hawaii, USA	*B*. *oleracea*	–	MK189265
A1839	UC 836.1	*Pectobacterium* sp.			–	MK189266
A5351	M6	*Pectobacterium* sp.		*Aglaonema* sp.	–	MK189267
A5358	J9	*Pantoea* sp.	Hawaii, USA	*Carica papaya*	–	MK182848
A3275	A811-1	*Pseudomonas* sp.	Hawaii, USA		–	MK202804
A4683	LGH5'	*Pseudomonas* sp.	Hawaii, USA	*B*. *oleracea*	–	MK202805
A3450	30	*Ralstonia solanacearum*	Trinidad	*S*. *lycopersicum*	–	MK242381
A3480	K350/XVT20	*Xanthomonas euvesicatoria*	Taiwan	*S*. *lycopersicum*	–	MG847376
A1696	K613/B-71	*X*. *vesicatoria*	California, USA	*S*. *lycopersicum*	–	MG847409
		Healthy potato			–	

Negative (-) sign indicates no amplification.

Healthy, greenhouse grown potato plants (~4 weeks old) were inoculated with four *D*. *dianthicola* isolates: PL22, PL24, PL25 and PL31. Stems of each seedling were stab inoculated with a sterile scalpel dipped in inoculum. Plants were kept in the greenhouse for three days; plants showing a black leg symptom on stems were collected for DNA extraction. Additional inoculations of tubers were completed with A5278 (*P*. *carotovorum* subsp. *carotovorum*) and A6152 (*P*. *carotovorum* subsp. *brasiliensis*). Briefly described, tubers were surface sterilized with 10% sodium hypochlorite (NaOCl) for three minutes, washed three times with sterile water, and then cut into slices. Slices were placed on filter paper (moistened with 5 ml sterile water) in petri dishes and stabbed with sterile toothpicks dipped in *Pectobacterium* sp. inoculum. Petri dishes were incubated at 28°C for 24 hours. Tubers exhibiting maceration symptoms were selected for DNA extraction.

DNA was extracted from pure bacterial colonies; healthy potato stems and tubers; naturally *D*. *dianthicola* infected potato plants; and artificially *D*. *dianthicola* and *Pectobacterium* sp. infected potato plants and tubers, respectively. Genomic DNA of all bacterial strains in the inclusivity and exclusivity panels were extracted using the DNeasy Blood and Tissue Kit (Qiagen, Germantown, MA) following the manufacturer’s instructions. DNA from infected and non-infected potato plant tissues were extracted using the Wizard Genomic DNA Purification Kit (Promega, Madison, WI). The manufacturer’s instructions were followed with an additional step of using a Mini-Bead Beater 16 Center Bolt (Biospec products, Bartlesville, OK) for one minute at maximum speed to thoroughly rupture cells. Following extraction, the NanoDrop 2000/c Spectrophotometer (Thermo Fisher Scientific, Waltham, MA) was used to estimate the DNA concentration of all samples (pure cultures and tissue).

### PCR and identity confirmation

*dnaA* and 16s rRNA gene regions were used for the identification of bacterial strains. 16s rRNA primers were selected from Dobhal et al. [22] to amplify 16s rRNA gene regions. Primers from Schneider et al. [38] were used to amplify the *dnaA* gene region. *Dickeya* sp. *dnaA* primer PCR conditions were: initial denaturation at 95°C at 5 min followed by 35 cycles of denaturation at 95°C at 20 sec, annealing 53°C at 60 sec, extension 72°C at 1 min, and final extension at 72°C at 2 min. *Pectobacterium* sp. and *R*. *solanacearum dnaA* primer PCR conditions were: initial denaturation at 94°C for 5 min followed by 35 cycles of denaturation at 94°C for 30 sec, annealing 61°C for 1 min, extension 72°C for 30 seconds, and final extension at 72°C for 4 min. *Xanthomonas* sp. *dnaA* primer conditions were: initial denaturation at 94°C for 5 min followed by 35 cycles of denaturation at 94°C for 30 sec, annealing at 61°C for 1 min, extension at 72°C for 30 sec, and final extension at 72°C for 10 mins. The 16s rRNA PCR conditions were followed as described by Dobhal et al. [22]. All PCRs were performed in the BIORAD T100 Thermocycler (Bio-Rad, Hercules, California). PCR products were electrophoresed on 1.5% agarose gel and the bands were visualized under FOTO/UV 26 (US PATENT 5347342) gel doc assembly. To clean the PCR products, 2 μl ExoSAP (Affymetrix Inc, Santa Clara, CA) was added with 5 μl of PCR product; incubated at 37°C for 15 min followed by 80°C for 15 min to deactivate the enzyme. The Sanger sequencing was performed at GENEWIZ facility, La Jolla, CA. The forward and reverse sequences were aligned using Geneious 10.2.3 software and evaluated manually for errors. Consensus sequences were obtained, and the identity was confirmed by searching the NCBI GenBank nucleotide and genome databases using the BLASTn tool. Multiple alignments of consensus sequences from the exclusivity and inclusivity panels were performed using Geneious.

### Target selection, primer design, and *in silico* validation

**Fig 1:**
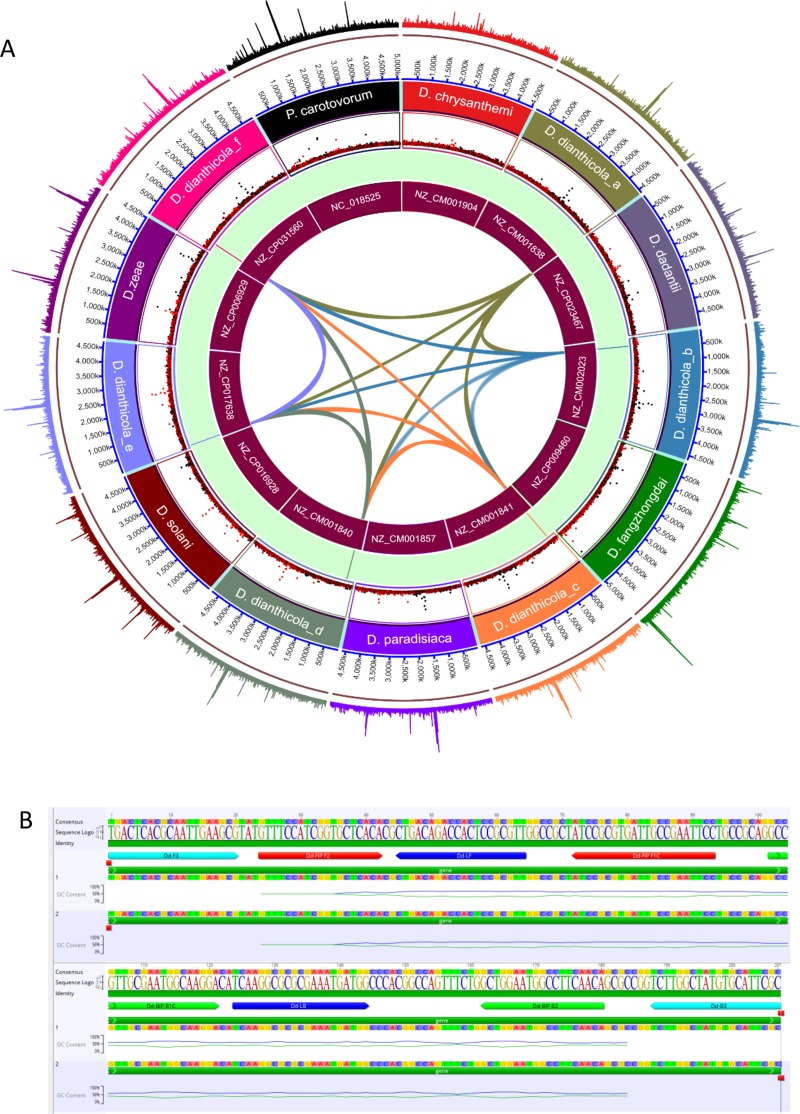
Genome alignment, unique gene alcohol dehydrogenase and primer locations. (A) Diagrammatic circa plot showing the presence of the target gene alcohol dehydrogenase used in the primer design for the *Dickeya dianthicola* specific LAMP assay. From the outermost circle to the innermost the circa plot displays: length of genomes in kilobases; name of the strains; lines in the green background depicts the presence of the target gene in *D*. *dianthicola* genomes; NCBI GenBank accession numbers for each genome used in the figure. The ribbons at the center of the circle represent the connections of the unique target gene among six strains of *D*. *dianthicola* (B) Location of all six LAMP primers and their orientations.

The alcohol dehydrogenase gene was determined as a unique region in *D*. *dianthicola* through genomic comparison of *D*. *dianthicola* (NZ_CM001838, NZ_CM001840, NZ_CM001841 and NZ_CM002023) with *D*. *chrysanthemi* (NZ_CM001904), *D*. *dadantii* (CP002038/NC_014500), *D*. *dianthicola* (NZ_CM001838, NZ_CM001840), *D*. *fangzhongdai* (NZ_CP025003), *D*. *paradisiaca* (CP001654), *D*. *solani* (NZ_CP009460, NZ_CP015137) and *D*. *zeae* (NZ_CP006929, NC_013592) (Dobhal and Arif, unpublished information). This unique gene was used to design LAMP primers for specific detection of *D*. *dianthicola* (Fig 1). Forward inner primer (FIP), forward outer primer (F3), backward inner primer (BIP), backward outer primer (B3), forward loop primer (LF) and backward loop primer (LB) were designed using PrimerExplorer V5 (https://primerexplorer.jp/e/). Specificity of each primer was verified by comparing the primer sequences against the NCBI GenBank nucleotide and genome databases using BLASTn tool. Primers were checked for possible secondary structures using MFOLD (http://unafold.rna.albany.edu/?q=mfold). Primer information is provided in Table 3.

**Table 3:** Details of LAMP primers designed using unique gene alcohol dehydrogenase for specific detection of *Dickeya dianthicola*.

Primer name	Sequence (5'-3')	Length (nt)	GC (%)	3'ΔG
Dd-FIP	GGAATTCGGCAATCACGCGGATGTTTCCATCGGTGCTCACA	41	54	-6.42	
Dd-BIP	GCCGTTGCGAATGGCAAGGATGTTGAAGGCCATTCCAGC	39	56	-4.86	
Dd-F3	TGACTCACGCAATTGAAGCG	20	50	-6.03
Dd-B3	GCGAATGCACATAGCCAAGA	20	50	-4.86
Dd-LF	AACGCGGAGTGGTCTGTCAG	20	60	-4.91
Dd-LB	TCAAGGCGCGCGAAATGATGG	21	57	-4.91

### LAMP assay specificity determination

The specificity of the developed LAMP primers was tested with a total of 16 *D*. *dianthicola* strains and 56 other strains included in inclusivity and exclusivity panels, respectively (Tables 1 and 2). DNA from soil and healthy plants were used as negative controls and sterile distilled water (molecular grade) served as non-template control (NTC). Three pairs of primer were used in the LAMP reaction, inner (FIP and BIP), outer (F3 and B3), and internal (LF and LB) primers, targeted the alcohol dehydrogenase gene (Table 3). LAMP reactions were completed in a 25 μl mix consisting of 15 μl isothermal master mix (Optigene; ISO-001), 2 μl LAMP primer mix (1.6 μM each of Dd-FIP and Dd-BIP, 0.2 μM each of Dd-F3 and Dd-B3, 0.4 μM each of Dd-LF and Dd-LB), 7 μl water, and 1 μl template DNA. LAMP reactions were carried out in the Rotor-Gene qPCR machine (Qiagen). Amplification at 65°C for 20 minutes followed by a melt curve analysis at 80–99°C with an increment of 0.2°C/sec. Melt curve graphs will show amplification above a threshold for positive reactions and no amplification below a threshold for negative reactions. After LAMP reactions were completed, results were also visualized by adding 3 μl of SYBR Green I (1:9 dilution) (Life Technologies Corporation, Eugene, OR). A positive reaction was indicated by a change in LAMP product color from orange to bright green, while negative reactions remained orange. Two percent agarose gel was used to run LAMP products for 90 minutes. After electrophoresis completion, bands were visualized under UV light.

### Limit of detection determination

Four tests were completed to determine the limit of detection: 10-fold serially diluted bacterial culture before DNA purification, 10-fold serially diluted purified genomic DNA, 10-fold serially diluted purified genomic DNA mixed with host DNA, and a 10-fold serially diluted synthetic DNA fragment. For 1^st^ LOD assay, overnight grown culture of *D*. *dianthicola* (1X10^9^ CFU/ml) which was confirmed by plating the serial dilutions prepared in 0.1% (w/v) peptone water (BBL, Becton Dickinson, Sparks, MD) on TZC media [22] and incubating the plates at 28°C for 12–18 h. The cell counts were recorded and calculated in terms of log10 CFU/ml. Serial dilutions were made from 10^8^ CFU/ml to 1 CFU/ml. Each of this concentration was mixed with 100 mg of the healthy plant tissues (potato stem) and DNA extraction was performed using the Qiagen DNeasy Plant Mini Kit following the manufacturer’s instructions. The LAMP assay was performed as described previously. For 2^nd^ LOD assay, purified genomic DNA of *D*. *dianthicola* strain A5573 was 10-fold serially diluted with water from 1 ng to 1 fg and used to perform the LAMP assay. For 3rd LOD assay, healthy potato tuber genomic DNA was added to each 10-fold serially diluted *D*. *dianthicola* strain A5573 purified genomic DNA during reaction preparation. This is referred to as a spiked test and was completed to observe any cross reactions with host material. For 4^th^ LOD assay, a synthetic DNA fragment was developed from Genewiz to confirm the LOD with higher accuracy. The synthetic DNA fragment was designed as mentioned by Arif et al. [39]. As with the purified genomic DNA, the fragments were 10-fold serially diluted (10^9^ to 10^1^ copies) with water. Non-template controls were included in each LAMP assay.

### Field applicability

DNA was extracted from greenhouse grown potatoes artificially infected with *D*. *dianthicola* strains (PL22, PL24, PL25 and PL31) using Plant Material Lysis Kits (Optigene, West Sussex, UK) as per the manufacturer’s instructions. Genomic DNA of *D*. *dianthicola* strain (PL22) was used as a positive control. LAMP reactions were prepared as previously mentioned. The amount of crude DNA added to reactions was 5 μl (instead of 1 μl) to 20 μl of LAMP reaction mixture containing 15 μl isothermal master mix, 2 μl LAMP primer mix (1.6 μM each of Dd-FIP and Dd-BIP, 0.2 μM each of Dd-F3 and Dd-B3, 0.4 μM each of Dd-LF and Dd-LB), and 3 μl water. Reaction tubes were prepared in two sets to compare the results. One set of tubes were incubated in a heating block (65°C) for 20 minutes and the other set of tubes were incubated in the Rotor-Gene qPCR machine under the same conditions. Immediately after incubation, 3 μl of SYBR Green I (1:9 dilution) were added to each reaction tube for both sets. Results were viewed under UV light. Products were electrophoresed (2% agarose gel for 90 minutes) and bands were observed under FOTO/UV 26 gel doc system.

### Multi-operator validation tests

Robustness of the developed LAMP assay was validated by a multi-operator test. Three operators independently performed the assay following the developed protocol. Each operator completed a blind test with six samples (three *D*. *dianthicola* isolates (PL23, PL24 and PL25), and three isolates (A5582, A5150 and A6159) from exclusivity panel, and non-template control).

## Results

### Primer design and *in silico* specificity

Six LAMP primers were designed with PrimerExplorer V5 using the alcohol dehydrogenase gene. Whole genomes of *D*. *dianthicola* and other closely related bacteria were aligned to identify alcohol dehydrogenase as the uniquely present genomic region in *D*. *dianthicola* (Dobhal and Arif, unpublished information). Using the NCBI GenBank BLASTn tool, primers showed 100% identity with 100% query coverage for *D*. *dianthicola* strains only (Table 3).

### Isolate identity confirmation

The detailed description of 16 bacterial strains used in inclusivity panel and 56 strains used in the exclusivity panel for developing an accurate LAMP diagnostic for *D*. *dianthicola* is presented in Tables 1 and 2, respectively. Identity confirmation of *D*. *dianthicola* and other bacterial strains were done by sequencing the sense and antisense strands using forward and reverse primers. Isolate identities were confirmed using the NCBI BLASTn tool. The accession numbers, MK189263—MK189274, MK243480—MK243481, and MK202803 –MK202805, of the submitted sequences were obtained during this study and are presented in Tables 1 and 2. Other accession numbers presented in Tables 1 and 2 were obtained during the other studies in the lab.

### LAMP assay specificity determination

**Fig 2:**
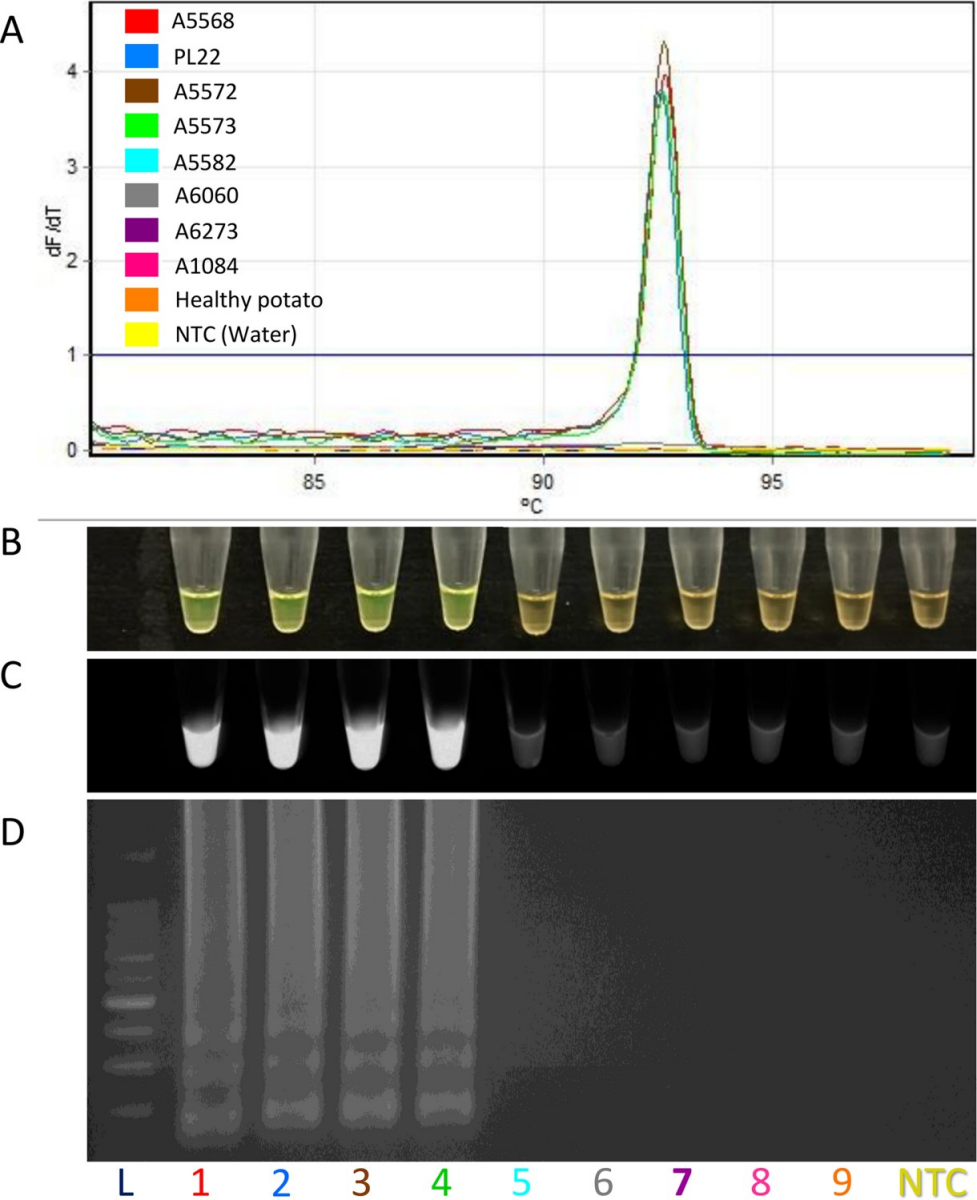
Specificity determination of LAMP assay developed for detection of *Dickeya dianthicola*. In this figure, four strains (1–4; A5568, PL22, A5572 and A5573) form inclusivity panel and four (5–8; A5582, A6060, A6273 and A1084) from exclusivity panel are included. **(A)** Melt curve diagram–only four strains of *D*. *dianthicola* were amplified, no melt curve was observed with non-*D*. *dianthicola* strains and negative controls. (**B)** Visualization of LAMP products after adding 3 μL of SYBR Green I stain in amplified LAMP products; green color represents positive amplification. **(C)** Visualization of SYBR Green I results under UV light; fluorescence indicative of positive amplification. **(D)** Agarose gel electrophoresis of the LAMP products on 2% agarose gel. L, 10 kb DNA molecular weight marker; 1, *D*. *dianthicola* (A5568); 2, *D*. *dianthicola* (PL22); 3, *D*. *dianthicola* (A5572); 4, *D*. *dianthicola* (A5573); 5, *D*. *solani* (A5582); 6, *D*. *dadantii* (A6060); 7, *P*. *carotovorum* subsp. *carotovorum* (A6273); 8, *Erwinia amylovora* (A1084); 9, healthy leaf potato (negative control); NTC, non-template control (water).

Specificity of the designed primers was assessed using 16 different *D*. *dianthicola* strains isolated from distinct geographic locations (Table 1). Additionally, specificity was tested with the exclusivity panel consisting of 56 other bacterial strains of *Dickeya* sp., *Pectobacterium* sp., other potato pathogens and saprophytes, and distant relatives (Table 2). All 16 *D*. *dianthicola* strains in the inclusivity panel were amplified by the LAMP assay (Table 1). Conversely, no amplification was observed for bacterial DNA from the exclusivity panel (Table 2). Results for inclusivity and exclusivity panel strains were confirmed using three different strategies (Fig 2). The first approach included a qPCR thermocycler-based fluorescence measurement and melting curve analysis. Fig 2A depicted the specific melting curves observed in real-time qPCR with four strains of *D*. *dianthicola*. The melting temperature among all *D*. *dianthicola* strains was about 92.5°C. The *D*. *dianthicola* melting curves were characterized by high peaks of 92.66°C, 92.56°C, 92.64°C and 92.60°C for the strains A5568, PL22 A5572, and A5573, respectively. No melting curve was observed for non-target bacterial strains. Furthermore, no other melting curve below the mean temperature (92.5°C) was observed, indicated that non-specific products were not present. Thus, the designed primers were highly specific and did not form non-specific products and/or primer-dimers. The second approach for amplification confirmation was a colorimetric based detection (Fig 2B). In this procedure, SYBR Green I was added to each tube after reaction completion. Only LAMP positive amplification turned bright green from orange and displayed fluorescence under UV light (Fig 2C). In contrast, non-amplified samples produced an orange color with no fluorescence. Finally, the third approach was electrophoresis; a 2% agarose gel stained with ethidium bromide was used to electrophorese the LAMP product. Positive amplification was indicated by the presence of a smear-like pattern (Fig 2D). All strains of the exclusivity panel showed no smear or band pattern on the gel, indicating no amplification. During all three confirmation tests neither healthy potato leaf tissue DNA nor the non-template control (NTC; water control) exhibited positive amplification.

### Specificity with naturally and artificially infected samples

**Fig 3:**
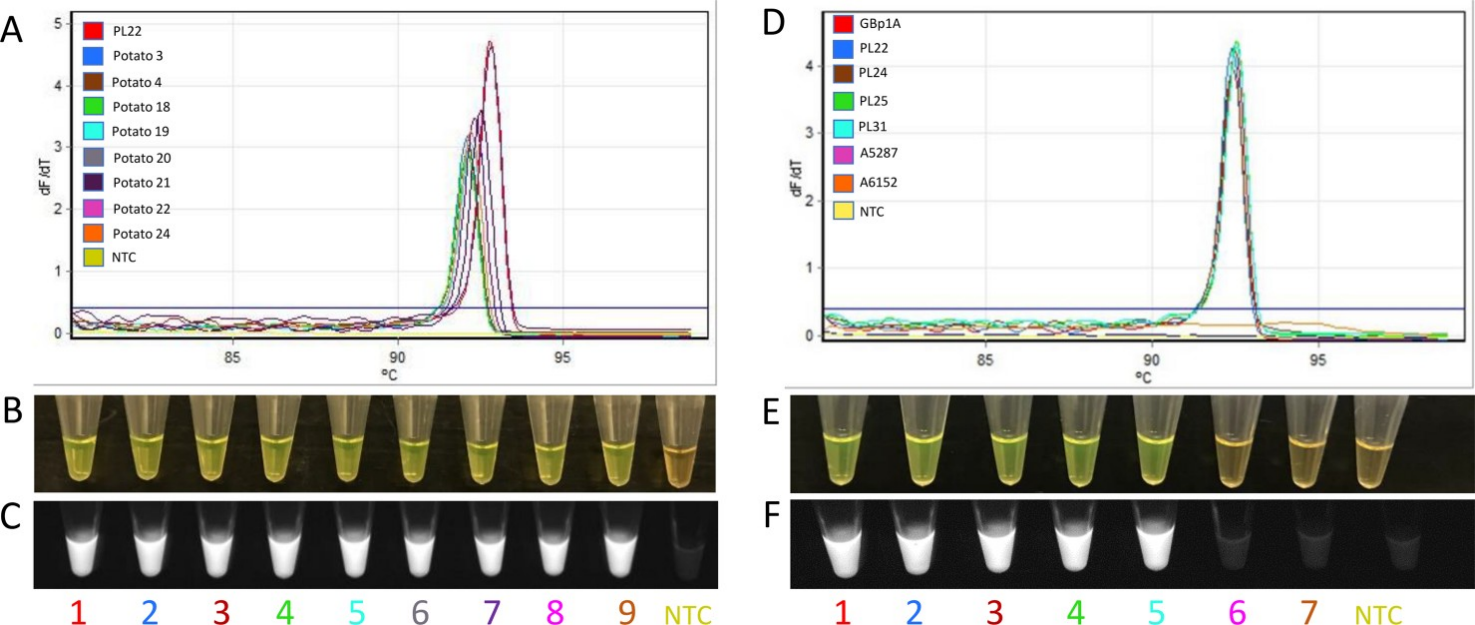
*Dickeya dianthicola* LAMP assay specificity determination with naturally and artificially infected plant samples. **A-C** LAMP assay results with eight naturally infected plant samples (2–9): **(A)** Melt curve represents the positive amplification; **(B)** visualization of LAMP products after addition of SYBR Green I stain, green color represents positive amplification; **(C)** visualization of SYBR Green I results under UV light exposure, fluorescence indicative of positive amplification. **D-F** LAMP assay results with artificially infected plant tissue samples. GBp1A (1)—positive control; PL22, PL24, PL25 and PL31 (2–5) are *D*. *dianthicola* infected plant samples; A5287 and A6152 (6–7) are *Pectobacterium* sp. infected plant samples; **(D)** Melt curve results; **(E)** visualization of LAMP products after addition of SYBR Green I stain, green color represents positive amplification; **(F)** visualization of SYBR Green I results under UV light exposure, fluorescence shows positive amplification. NTC is non-template control (water); No false positives or false negatives were observed.

The LAMP assay was evaluated using *D*. *dianthicola* DNA extracted from naturally and artificially infected potato plants. Additionally, *P*. *carotovorum* subsp. *carotovorum* and *P*. *carotovorum* subsp. *brasiliense* DNA isolated from artificially infected potato plants were also used. DNA extracted from *D*. *dianthicola* infected plants produced a melt curve and a color change from orange to bright green after the addition of SYBR Green I, and fluorescence under UV light. On the other hand, potato plants artificially infected with *Pectobacterium* sp. did not produce any positive results (Fig 3). No amplification was observed in non-template control and healthy potato DNA. No false positives or false negatives were observed.

### Limit of detection determination

**Fig 4:**
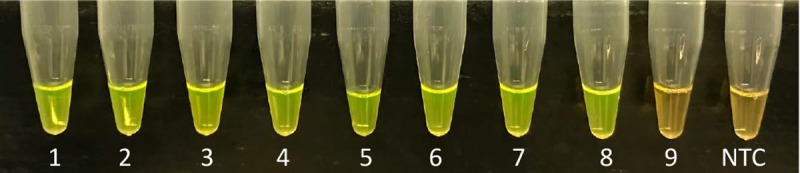
Determination of LAMP assay’s limit of detection using 10-fold serially diluted pure culture of *Dickeya dianthicola*. LAMP product visualized after addition of SYBR Green I stain, positive amplification turned orange color to bright green. Tubes 1–9 showed the detection from 10^8^ CFU/ml to 1 CFU/ml; NTC is non-template control. Results showed positive amplification down to 10 CFU/ml.

**Fig 5:**
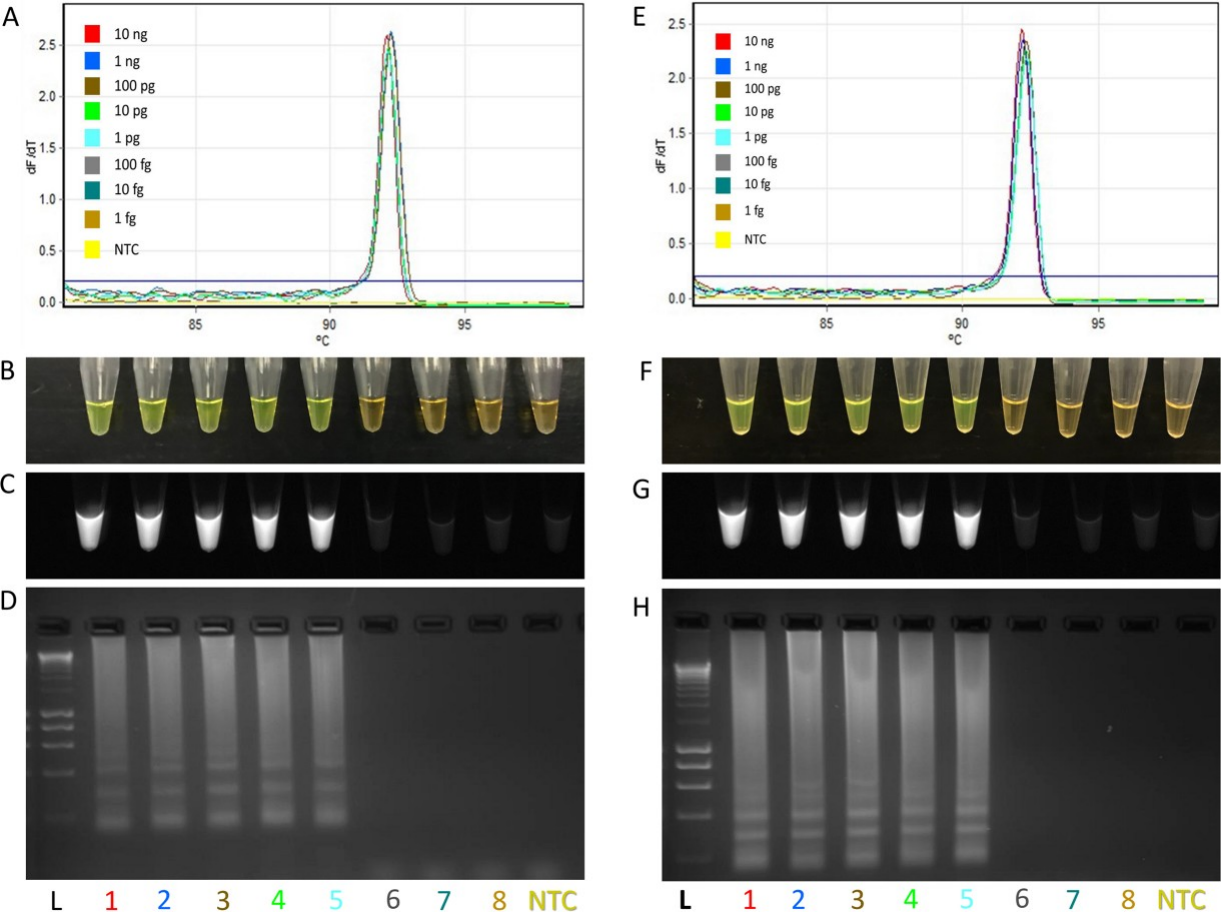
Determination of limit of detection of *Dickeya dianthicola* LAMP assay using 10-fold serially diluted genomic DNA. **A-D**: Sensitivity of serially diluted *D*. *dianthicola* genomic DNA (L = 100 bp DNA ladder, 1 = 10 ng, 2 = 1 ng, 3 = 100 pg, 4 = 10 pg, 5 = 1 pg, 6 = 100 fg, 7 = 10 fg, 8 = 1 fg, NTC = non-template control. **E-H**: Sensitivity assay of serially diluted *D*. *dianthicola* genomic DNA spiked with potato genomic DNA (L = ladder, 1 = 10 ng, 2 = 1 ng, 3 = 100 pg, 4 = 10 pg, 5 = 1 pg, 6 = 100 fg, 7 = 10 fg, 8 = 1 fg, NTC = non-template control). **(A, E)** Melt curve of sensitivity assay; **(B, F)** LAMP product visualized after addition of SYBR Green I stain, positive amplification turned orange color to bright green color; **(C, G)** LAMP product with SYBR Green I stain under UV light, fluorescence indicated positive amplification; **(D, H)** agarose gel electrophoresis of LAMP product on 2% agarose gel.

LOD or sensitivity of the developed assay was determined using a 10-fold serially diluted pure culture of *D*. *dianthicola* before DNA isolation; LOD was 10 CFU/ml (Fig 4). Other LOD experiments were performed from 10-fold serially diluted *D*. *dianthicola* genomic DNA or synthetic DNA; assays were performed three times; each time, the detection limit was 1 pg. Additionally, spiked assays were performed by adding 1 μl of healthy potato genomic DNA into the LAMP reaction containing 10-fold serially diluted *D*. *dianthicola* genomic DNA to confirm the inhibitory or background effect of the host genomic DNA. The spiked assays also detected 1 pg of *D*. *dianthicola* DNA; host DNA did not show any adverse effect on LAMP assay performance (Fig 5). LOD assay was also performed using the 10-fold serially diluted synthetic DNA fragment containing the primer target sites to confirm the detection limit; the assays detected down to 1,000 copies (S1 Fig). NTC was included in each sensitivity assay.

### Field applicability

**Fig 6:**
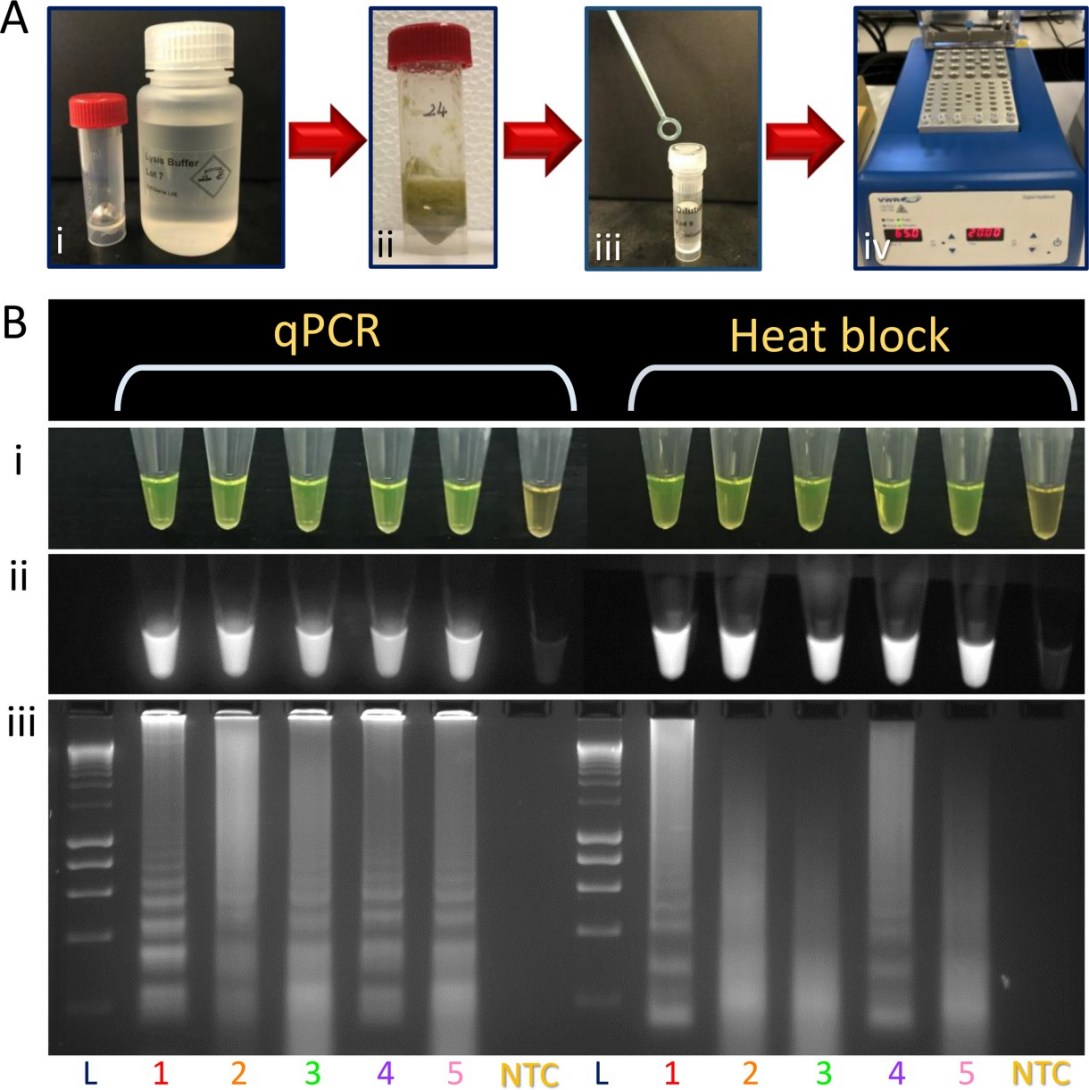
Validation of field applicability of *Dickeya dianthicola* specific LAMP assay by comparing the LAMP results using real-time qPCR and heat block. (**A)** Flow diagram of the DNA extraction process of naturally infected plant samples by using the plant material lysis kits: **i**—plant material was processed in a tube containing iron ball and 1 mL lysis buffer; **ii***–*macerated plant tissue after shaking vigorously for 1 min; **iii–**loop full of macerated supernatant was transferred to new vial containing dilution buffer; **iv**—five μL of diluted sample (crude DNA template) was added to LAMP assay and reaction was incubated at 65°C in a heat block for 20 minutes. **(B)** Visualization of LAMP products amplified using real-time qPCR machine and heat block: **i**–visualization after addition of SYBR Green I, bright green indicated positive amplification; **ii**—visualization after addition of SYBR Green I under UV, fluorescence indicated positive amplification; **iii–**LAMP products were electrophoresed on 2% agarose gel and visualized under UV, smear-like pattern reflected positive amplification. L, DNA molecular marker; 1, Genomic DNA of *D*. *dianthicola* (PL22, positive control); 2, *D*. *dianthicola* infected; 3, *D*. *dianthicola* infected; 4, *D*. *dianthicola* infected; 5, *D*. *dianthicola* infected; NTC, non-template control (water).

DNA was extracted from *D*. *dianthicola* infected, greenhouse-grown potato plants using a completely field-deployable plant material lysis kit. The LAMP reactions were incubated in a heating block at 65°C. The obtained results were in 100% agreement to the results observed using a real-time qPCR machine. Results were reproducible and obtained in 20 minutes (Fig 6). Visualization of LAMP products with SYBR Green I revealed product color change to bright green (*D*. *dianthicola;* positive) or orange (negative). NTC was included in each run; no false positives or negatives were detected.

### Multi-operator validation tests

Multi-operator validation tests were performed by three different and independent operators with a blind panel of six different samples to confirm the reproducibility and robustness of the developed LAMP assay. The samples consisted of genomic DNA from *D*. *dianthicola* strains (PL23, PL24 and PL25), *D*. *solani* (A5582), *P*. *parmentieri* (A6159) and, *E*. *cloacae* (A5150). Non-template control was included with the six samples as negative control. The obtained results from all three operators were in 100% agreement with previously obtained results. No false positives or false negatives were detected.

## Discussion

We have developed a LAMP assay that is rapid, sensitive, and specific for detection of *D*. *dianthicola*. This phytopathogen is of great concern because it is highly quarantined against and causes the destructive disease, blackleg of potato. Other molecular techniques have been developed for specific detection of *D*. *dianthicola*, but lack specificity or portability [40, 41]. Our LAMP assay has shown to be not only in-field usable, but also rapid, which is important for produce and plants that are time-sensitive commodities.

Specificity of the LAMP assay was tested using strains present in inclusivity (16 *D*. *dianthicola*) and exclusivity (56 other bacteria) panels. Analysis of the melt curve obtained in the qPCR displayed homogeneous melt peaks around 92.5°C exclusively for *D*. *dianthicola* while no melting curves were observed for the non-target bacterial strains (Fig 2A). Additionally, no irregular curves formed below the mean temperature (92.5°C) suggesting a lack of primer-dimer formation or cross-reaction with other targets, demonstrating the high specificity of our primers. The LAMP assay amplified all *D*. *dianthicola* strains (Table 1) and detected no amplification of non-target bacteria (Table 2), indicating high assay specificity. Other molecular detection methods exist for *Dickeya* sp. [37, 41], but either lack specificity or were not tested for field applicability. In another assay, developed primers detected all *D*. *dianthicola* strains, but only *Dickeya* isolates were used for testing [40]. We incorporated closely related genera such as *Pectobacterium* and other potato pathogens to ensure that developed LAMP assay was exclusive of bacteria with similar genes or genomes. Moreover, no field-deployable LAMP assay exists for the specific detection of *D*. *dianthicola*.

Consequently, the inclusivity and exclusivity panels indirectly confirmed the target, alcohol dehydrogenase gene, as unique to *D*. *dianthicola*. This unique gene was identified by performing comparative whole genome analyses of *D*. *dianthicola*, *Dickeya* species, and other closely related genera (Dobhal and Arif, unpublished information). In TaqMan qPCR assay, the authors targeted the *dnaX* gene, but were not able to detect all target *D*. *dianthicola* strains [41]; also, *dnaX* gene is not completely specific to *D*. *dianthicola*. Additionally, a study developed a LAMP assay targeting a region of the *mglC* gene, but the assay was limited to detection of the genus *Dickeya* [37]. Identifying a unique target sequence is imperative to developing a robust and highly specific assay [22, 23].

Field-testing was completed with field-deployable DNA extraction kits and a heat block (65°C). When compared to LAMP reactions incubated in a qPCR machine under specificity panel conditions, results were 100% comparable within 20 minutes when SYBR Green I stain was added for visualization of amplification products (Fig 6). Additional visualization under UV light and through gel electrophoresis confirmed accurate amplification using the heat block. Consequently, we demonstrated that developed LAMP assay equipment could be simplified to a steady heat source and can be performed in field. Similarly, Larrea-Sarmiento et al. [23] reduced complexity and portability through use of the field-deployable, portable BioRanger that detected target bacteria within 15 minutes. In contrast, other developed molecular detection techniques [40, 41] are time consuming and require complex tools. Reducing complexity and time is important for use by any operator at point-of-care sites. Moreover, simplifying the machinery adds to the cost-effectiveness of the protocol.

LOD of LAMP assay was confirmed by performing four independent sensitivity tests: 1) 10-fold serially diluted *D*. *dianthicola* pure culture; 2) 10-fold serially diluted *D*. *dianthicola* DNA; 2) 10-fold serially diluted *D*. *dianthicola* DNA spiked with host DNA; and 3) 10-fold serially diluted synthesized DNA fragment (Figs 4 and 5 and S1). The limit of detection for sensitivity and spiked sensitivity tests were consistent to 1 pg and for synthesized targets up to 1,000 copies (Figs 5 and S1). However, Yasuhara-Bell et al. [37] had a detection limit of 5 pg for *Dickeya* sp. and detection time varied depending on the type of sample DNA (purified, cultured, or crude). Detection using the 10-fold serially diluted cells followed by DNA isolation, showed high sensitivity (10 CFU/ml) compared to the sensitivity performed using 10-fold serially diluted genomic DNA—the lower LOD could be the result of quantification method used, that is NanoDrop. NanoDrop does not provide precise quantification of the double stranded DNA, and we have experienced this in our lab. But the method used to determine the CFU/ml was very accurate since colonies from each dilution were recalculated on media plate. Before DNA isolation from each dilution, 100 mg of plant tissues were added to mimic as the real infected sample. In our study, three operators independently performed the LAMP assay with unknown samples; all operators produced concordant results, confirming the high robustness of the developed LAMP assay.

LAMP assays are comparatively prone to cross contamination because of the high number of copies produced during amplification. However, contamination can be reduced by adding the detection dye before the reaction starts or devising a method to release the dye after reaction completion [42]. Consequently, including the detection dye in a prepared reaction tube reduces complexity as well as increases in-field usability and portability.

Here we have demonstrated that LAMP assays can be simplified to 3 steps: DNA extraction with Optigene DNA purification kit, incubation in a heat block, and addition of detection dye for visualization (Fig 6). This feature of LAMP is convenient in low-resource field situations where conventional DNA or RNA extraction prior to diagnostic testing is impossible. Our LAMP assay lays the groundwork for not only *D*. *dianthicola* diagnostics, but also for other pathogens. Ultimately, the developed detection assay can be incorporated in diagnostics for securing our borders against phytopathogens that threaten food security and economies worldwide.

## Supporting information

S1 Fig Sensitivity validation of *Dickeya dianthicola* specific loop-mediated isothermal amplification (LAMP) using synthetic DNA fragment containing the primer target sites. Ten-fold serially diluted synthetic DNA fragment was added from 10^9^ to 10^1^ copies number per reaction. Number of copies per reaction are indicated at the bottom of the figure. L–ladder and NTC–non-template control. **(A)** Sigmoid curve indicated the positive amplification and detected up to 10^3^ copies; **(B)** LAMP products after addition of 3 μL of SYBR Green I stain in each tube; green color indicated positive amplification; **(C)** LAMP products with SYBR Green I stain under UV light; fluorescence indicated positive amplification; **(D)** LAMP products electrophoresed on a 2% agarose gel and visualized under UV.
(TIF)
