# Supplementary figures and images for: Development of a robust, field-deployable loop-mediated isothermal amplification (LAMP) assay for specific detection of potato pathogen Dickeya dianthicola targeting a unique genomic region
Source: PLoS One. 2019 Jun 24;14(6):e0218868. doi: 10.1371/journal.pone.0218868 (PMC6590888; doi:10.1371/journal.pone.0218868)

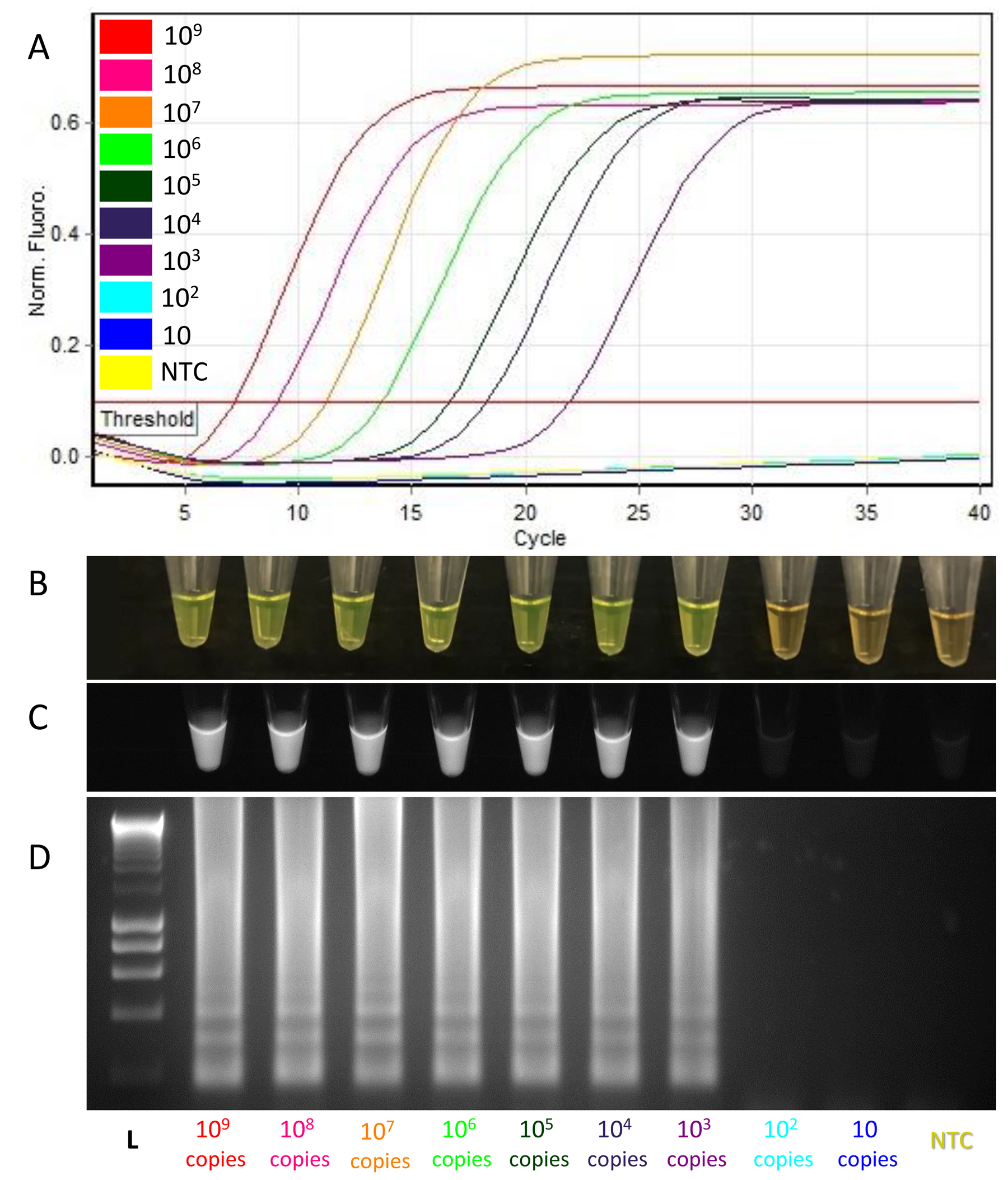

Supplement: S1 Fig — Ten-fold serially diluted synthetic DNA fragment was added from 109 to 101 copies number per reaction. Number of copies per reaction are indicated at the bottom of the figure. L–ladder and NTC–non-template control. (A) Sigmoid curve indicated the positive amplification and detected up to 103 copies; (B) LAMP products after addition of 3 μL of SYBR Green I stain in each tube; green color indicated positive amplification; (C) LAMP products with SYBR Green I stain under UV light; fluorescence indicated positive amplification; (D) LAMP products electrophoresed on a 2% agarose gel and visualized under UV. (TIF) [file pone.0218868.s001.tif]
